# Outcomes of fixation of Vancouver B periprosthetic fractures around cemented versus uncemented stems

**DOI:** 10.1186/s12891-023-06359-0

**Published:** 2023-04-04

**Authors:** Ameen Barghi, Philip Hanna, Nelson Merchan, Michael J. Weaver, John Wixted, Paul Appleton, Edward Rodriguez

**Affiliations:** 1 Atrium Health Wake Forest Baptist Medical Center, Winston-Salem, NC USA; 2 Department of Orthopaedics, Beth Israel Deaconess Medical Center, 330 Brookline Ave, Stoneman 10, Boston, MA 02215 USA; 3 Harvard Medical School Orthopaedic Trauma Initiative, Harvard Medical School, Boston, MA USA; 4 Brigham and Women’s Hospital, Boston, MA USA

**Keywords:** ORIF, Press-fit stem, Vancouver B, Hip fracture, Geriatric trauma, Periprosthetic femur fracture

## Abstract

**Background:**

The incidence of periprosthetic femur fracture (PPFF) in the setting of total hip arthroplasty (THA) is steadily increasing. We seek to address whether there is a difference in outcomes between Vancouver B fracture types managed with ORIF when the original stem was a press-fit stem versus a cemented stem.

**Methods:**

In this retrospective cohort study at a level 1 trauma center, we identified 136 patients over 65 years-of-age with Vancouver B-type fractures sustained between 2005 and 2019. Patients were treated by ORIF and had either cemented or press-fit stems prior to their injury. Outcomes were subsidence of the femoral implant, time to full weight bearing, rate of the hip implant revision, estimated blood loss (EBL), postoperative complications, and the one-year mortality rate.

**Results:**

A total of 103 (75.7%) press-fit and 33 (24.3%) cemented patients were reviewed. Patient baseline characteristics, Vancouver fracture sub-types, and implant characteristics were not found to be significantly different between groups. The difference in subsidence rates, postoperative complications, and time to weight bearing were not significantly different between groups. EBL and one-year mortality rate were significantly higher in the cemented group.

**Conclusions:**

In geriatric patients with Vancouver B type periprosthetic fractures managed with ORIF, patients with an originally press fit stem may have lower mortality, lower estimated blood loss, and similar subsidence and hospital length of stays when compared to those with a cemented stem.

## Introduction

As the volume and indications for total hip arthroplasties (THAs) continue to grow, so does the prevalence of periprosthetic femur fractures (PPFF) [1–3]. The Vancouver system is a three-category system based on fracture location, implant stability, and quality of surrounding bone stock [4, 5]. Vancouver B-type PPFFs, specifically B2 fractures, are the most common type of PPFFs and most often occur in elderly patients with limited physiologic reserve [6, 7]. Traditionally, the management of a periprosthetic Vancouver B fractures with loose prostheses (B2 and B3) has involved revision arthroplasty (RA), while Vancouver B1 are commonly managed with fixation alone [8].

There is increasing evidence in the trauma literature that fixation of these types of fractures may offer similar outcomes to revision arthroplasty, particularly in older patients with decreased functional demand [9]. More recently, studies have found similar outcomes when managing B2 and B3 fractures with open reduction and internal fixation (ORIF) when compared to RA [9–11]. At our institution periprosthetic fractures, including all Vancouver B variants, are primarily treated with fixation and only revised if the stability of the original stem cannot be achieved. The rationale for this approach is that if stem stability is restored with the original stem in situ, then revision to a new stem may not be required, particularly in elderly patients with comorbidities or otherwise debilitated.

Management of B2 and B3 fractures with ORIF without revision inherently requires a stem design that can regain original stability after fracture reduction [12]. Cemented stems offer stability in the form of controlled subsidence within a cement mantle that offers stability upon cement polymerization [13]. Solomon et al. advocates for ORIF alone of cemented stems with periprosthetic fractures should the cement–bone interface be maintained and only the cement-stem interface disturbed by fracture [14]. Uncemented prostheses have been reported to have less risk of septic loosening and a higher union rate [15, 16]. In our experience, fractures around cemented implants are often more technically challenging to manage by ORIF, where both a bone-cement and cement-stem interface contribute to implant stability [17]. Patients with cemented implants also often have poor bone quality and may be ultimately require revision arthroplasty in the event of periprosthetic fracture with inability to restore stem stability with ORIF alone [18], which adds to the technical challenges of a case.

We seek to address if there is a difference in outcome between Vancouver B fracture types managed with ORIF when the original stem was a press-fit stem versus a cemented stem. We hypothesize that fixation of cemented stems, where the cement mantle may be disrupted, may not offer a durable restoration of stability even if fixation is initially successful, and may be best treated by RA instead of ORIF.

## Methods

This study was performed under approval from the Institutional Review Board (IRB) for ethical approval and informed consent requirements and all methods were carried out in accordance with relevant guidelines and regulations. The billing records were screened using current procedural terminology (CPT) code to identify patients treated for PPFF at a single institution between 2005 and 2019. Inclusion criteria were patients with PPFF around a hip implant classified as Vancouver type B, managed surgically at our institution with open reduction and internal fixation without revision of the hip implant. Patients with non-Vancouver variant PPFF and with PPFF around a hip implant classified as Vancouver A or C, patients treated with revision of the hip implant, patients who required more than 180 days to achieve full weight-bearing, and those with infection or nonunion at the time of first presentation were excluded.

Two hundred and sixty-three patients were identified. Patient records and preoperative radiographs were reviewed to determine the eligibility for inclusion. One hundred and forty-three patients met the inclusion criteria and underwent a chart review. Fractures were classified by the senior author (E.K.R) and confirmed by three other authors in a blinded fashion according to the Vancouver classification system using the preoperative radiographs as well as intraoperative reports of implant stability [19]. Information about the demographic characteristics, fracture classification, implant characteristics, Charlson Comorbidity Index (CCI), and the American Society of Anesthesiologists Classification (ASA) were collected. The patients were then divided into two groups according to their type of implant at the time of injury. There were 107 patients with press-fit and 36 patients with cemented implants. The primary outcome was the subsidence of femoral implant evaluated in the postoperative radiographs at 6, 12, 36, and 52 weeks after surgery using the tip of greater trochanter to the shoulder of the stem (TG-SS) calculated distance method, where a change from the immediate postoperative measurement of 3 mm or more was considered clinically significant [19]. Secondary outcomes included time to full weight bearing, rate of the hip implant revision, estimated blood loss (EBL), postoperative complications, and the one-year mortality rate.

The student t-Test was used to compare continuous variables and chi-square to compare categorical variables. Beside the rate of subsidence, we used the Wilcoxon test to compare the time-to-event between the two groups. A Cox-regression model was used to analyze the one-year mortality rate adjusting for baseline and surgical variables.

## Results

Eventually 136 patients were included in the analysis, 103 of them had a press-fit implant and 33 had a cemented implant at the time of injury. Four of those patients belong to the press-fit implant group and 3 to the cemented implant group, there was no significant difference between those patients regarding their baseline characteristics.

There were 59 fractures classified as Vancouver type B1, 40 as Vancouver type B2, and 37 as Vancouver type B3. There was no significant difference between the two groups considering the fracture type (*p* = 0.4079). There were 43 fractures around hemiarthroplasty implant, 81 around a primary total hip implant, and 12 around a revision total hip implant of which 2 had more than one revision procedures prior to the index fracture. There were 5 inter-prosthetic features with ipsilateral total knee replacement implant. None of the implant characteristics were found be significantly different between the two groups.

Patient demographic characteristics (age, gender and BMI) were not significantly different among the two groups. Basic demographic data is displayed in Table 1. Time from presentation to surgery was not different between the cemented and press-fit patients (1.8 and 1.6 days respectively). However, the estimated intraoperative blood loss was significantly higher in the cemented group 495 ± 432 cc vs 471 ± 288 cc (*p* = 0.0243).

**Table 1:** Demographic data

	Cemented	Press-fit	*P*-value
Age	83 ± 11.7	82 ± 11.3	0.747
Gender, Male (%)	13 (39.4%)	34 (33%)	0.502
BMI (kg/m^2^)	24.1 ± 5.6	26.1 ± 5.9	0.7512
ASA Class			0.119
1	1 (3%)	0	
2	6 (18.2%)	22 (21.4%)	
3	20 (60.6%)	73 (70.9%)	
4	6 (18.2%)	7 (6.8%)	
5	0	1 (0.97%)	
CCI			
	5 (4–6)	5 (4–6)	0.796653
Fracture Type			0.4079
B1	16 (48.5%)	43 (41.8%)	
B2	11 (33.3%)	29 (28.2%)	
B3	6 (18.2%)	31 (30%)	

Subsidence of the stem was found in 10 (12%) of the press-fit group vs 4 (17%) in the cemented during the first year after surgery, however the difference in the subsidence rate between the two groups was not statistically significant (*p* = 0.5171). Moreover, Wilcoxon time to event analysis revealed no statistically significant difference between the two groups (*p* = 0.5274).

When assessing time to full weight bearing (days), the cement group achieve it around 71.5 ± 48.8 vs 80 ± 41 in the press-fit group, this difference was not significant. Details of the secondary outcomes are displayed in Table 2.

**Table 2:** Cumulative outcomes

	Cemented	Press-fit	*p*-value
Time to Surgery (days)	1.8 ± 1.8	1.6 ± 2.2	0.1478
EBL (ml)	495 ± 432	471 ± 288	**0.0026**
Length of Stay (days)	6.4 ± 3.3	6.6 ± 4.7	**0.0243**
Revision Post-Fracture	0	3 (2.91%)	0.4313
Time to the full WB (days)	71.5 ± 48.8	80 ± 41	0.3042
Subsidence within one year	4 (17.4%)	10(12.2%)	0.5171

**Fig. 1:**
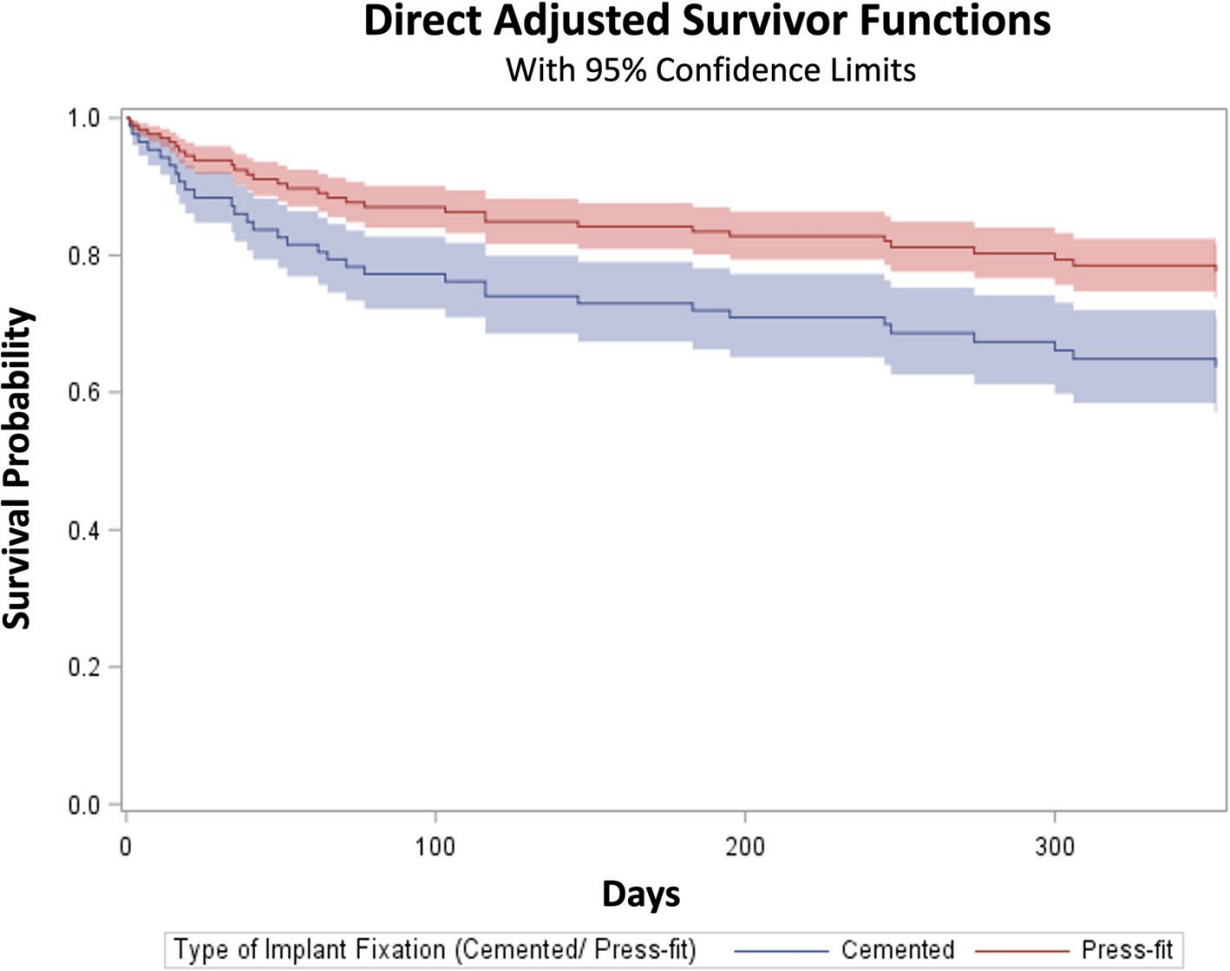
Press-fit vs. cemented group risk of death during the first postoperative year

We looked at the one-year mortality rate using the Cox Regression model with adjustment for sex, age, baseline ASA classification, length of stay, estimated blood loss and peri-surgery complication. Significant variables after stepwise model were included in Table 3. The press-fit group had significantly lower risk of death during the first postoperative year compared to the cemented group (hazard ration 0.49 [95% confidence Interval 0.33–0.71], *p* = 0.0002) (Fig. 1).

**Table 3:** Survival probability of cox-regression model within 1 year

	Hazard Ratio (95% CI)		*P*-value
Female	0.37	0.25–0.55	< 0.0001
Age (yrs)	1.09	1.07–1.11	< 0.0001
ASA class	3.32	2.33–4.74	< 0.0001
Press-Fit vs. cemented	0.49	0.33–0.71	0.0002
LOS (days)	1.09	1.02–1.13	0.0001

No perioperative complications were reported. There were 3 patients in the press-fit implant group who underwent a revision for the hip implant. One of those patients had an infected nonunion, another had a deep infection which didn’t respond to two debridement procedures, and the third had persistent pain due to implant loosening. Moreover, among the press-fit implant group, there was one patient suffered postoperative infection who responded well to one surgical debridement, one patient with painful bony exostosis, and one patient with persistent pain that couldn’t be correlated with infection of implant instability. There were no incidents of implant revision found in cemented implant group; however, there was one patient who received a revised fixation due to refracture.

## Discussion

The most important finding of this study is that in geriatric patients with Vancouver B type periprosthetic fractures managed with ORIF, patients with an originally press-fit stem may have lower mortality, lower estimated blood loss, and similar subsidence and hospital length of stays when compared to those with a cemented stem. The treatment of PPFFs is technically demanding, with high complication rates and often poor outcomes and high mortality [20]. The choice for treatment is contingent on fractures pattern, implant type and stability, and bone stock. The Vancouver classification system gives surgeons some algorithmic treatment guidance [21]. However, there is currently debate surrounding the recommendations for the proper treatment of Vancouver B type fractures, particularly B2 and B3 fractures. In their original 1995 paper describing the treatment algorithm that parallels the Vancouver classification, Duncan and Masri do not differentiate treatment branches should a patient’s implant be cemented or press-fit [21]. To our knowledge, this study is the first and largest to date that sought to determine whether there was a difference in outcomes after ORIF of Vancouver B type PPFFs in patients who had received cemented versus press-fit implant stems. In our study, there was not a difference between the study groups regarding the patients’ demographics, type of PPFF and implant characteristics. Our primary outcomes were subsidence of implants. Other outcomes include the need for subsequent revisions surgeries, time to full weight bearing, estimated blood loss (EBL), postoperative complications, and the one-year mortality rate. We originally hypothesized that the ORIF of cemented stems would not provide adequate stability, even in light of successful operative fixation. However, in this study, we found no statistically significant difference between the cemented implant and press-fit implant groups.

The rate of cemented and hybrid prostheses implants seems to be on the rise, while uncemented implants have been relatively stable [22, 23]. The National Joint Registry of England and Wales reported a 1.5- and 1.6- fold increase in periprosthetic fractures around these cemented implants between 2011 and 2016 [23]. In a review article, Quah et al. advocate for using the structural integrity of the cement mantle and good bone stock as a surrogate for the stability of the prosthesis alone to determine appropriate treatment of Vancouver B type fractures. They believe that ORIF with anatomic reduction is an appropriate option for B type fractures [24]. In a meta-analysis of 11 articles, Bennett et al. found that method of original fixation (cemented vs. press-fit) did not accurate predict Vancouver B1 or B2 fractures [25]. In a randomized controlled trial comparing functional and radiological outcomes of patients treated with cemented versus cementless stems after index femoral neck fractures, Barenius et al. found good function and few complications at four years follow-up. They do, however, report poorer short-term functional outcomes among cementless patients and caution against their routine use [26]. Conversely, Langslet et al. reported better Harris Hip Scores in patients with a cementless stem after five years. They also found 7.4% periprosthetic fractures in the cementless group when compared with 1% in the cemented group after five years [27]. These studies suggest little reason to believe that baseline characteristics ought to differ between these two groups.

To our knowledge, few studies have commented on the risk factors associated with outcomes after Vancouver B fractures are treated with ORIF. Zheng et al. found young age to be the only risk factor for complication in 97 patients with Vancouver B fractures. They did not find cemented implants to be of statistical significance [28]. In our study, none of the patients underwent ORIF in the cemented implant group underwent a subsequent revision versus three revisions reported in the press fit implant group. In a review of 116 patients, Springer et al. found that cemented revision components had higher re-revision rates when compared to fully and proximally coated revision cement-less components in Vancouver B fractures, though proximally coated implants had the highest complication rates. The most frequent long-term complications were prosthetic loosening and non-union. Results were improved when an uncemented, porous-coated stem was used [29]. In Smitham et al.’s cohort of 52 Vancouver B2 PPFFs with a cemented polished double-tapered components treated by ORIF, all fractures healed within the first three months [30].

Kristensen et al. used the Norweigian Hip Fracture Register to find that certain cemented femoral components, such as highly polished dual tapered stems, were associated with higher rates of PPFF when compared to anatomical and straight stems [31]. In a study of 70 uncemented hemiprostheses and 174 cemented hemiprostheses, Foster et al. found a higher percentage of iatrogenic and postoperative periprosthetic fractures in the uncemented group [32]. We found that estimated blood loss was significantly higher (~ 5%) in the cemented group than the press-fit group. Volume of blood lost and the need for transfusion have been cited as important markers of post-operative outcomes in frail and geriatric patients [33]. Stenvers et al. categorized 63 patients using the complex fracture frailty index and found that more minimally invasive surgeries, such as ORIF, when compared to more invasive revision arthroplasty resulted in more major complications (30-day, 90-day, and 1-year mortality) as well as minor complications (implant infections, pneumonia, blood transfusions, and urine tract infections) [34]. This data is particulalry applicable, as most Vanvouver PPFF patients tend to be geriatric patients.

For patients with complications, the length of hospital stay is significantly longer and hip function is significantly worse [35]. Illustrating accurate cost differences between cemented and press-fit patients in different healthcare systems is complex. Length of stay is a well-accepted proxy for ward costs. In a prospective study of 146 patients, Phillips et al. demonstrated that 80% of the cost of treatment for PPFF patients was derived from ward costs, while only 7% was the actual implant cost [36]. In our study, we found no difference in length of stay between our cemented and press-fit PPFFs treated with ORIF.

After adjusting for baseline characteristics, we found that our press-fit group had a significantly lower risk of mortality than the cemented group. Our observed difference in mortality may be a reflection of a frailer group among cemented patients. However, all baseline characteristics that were measured were statistically similar. In a 2010 Cochrane review of primary total hip arthroplasties with and without cement for femoral neck fractures, the authors concluded that cemented fixation resulted in less pain and improved mobility, but afforded no difference in complication rates or mortality [37]. In a more recent randomized controlled trial, Inngul et al. found that their cemented group performed better in all of their metric scores and they did not support the use of an uncemented stem for femoral neck fractures [38]. When analyzing claims data from the National Health Insurance Database and the National Register of Deaths Database, Tsai et al. found that cemented patients had a significantly higher mortality risk than non-cemented patients within 7, 30, 180 days and 1 year post-opereatively [39]. Alternatively, Dale et al. used the Norwegian Arthroplasty Register from 2005 to 2018 to study almost 80, 000 THAs and long-term mortality was similar regardless of fixation type [40].

In our cohorts, the rate of subsidence of the implants were similar and not significant between groups. Our press-fit group had a subsidence rate of 12%, which was similar to Ko et al.’s 17% subsidence in 12 geriatric patients [41]. In a prospective study of 22 Vancouver B2 and B3 patients with uncemented stems, Fink et al. found no incidences of subsidence after a two-year follow-up period [42].

Our study has several limitations. First, our retrospective design lacks a random control group. Second, several orthopaedic traumatologists participated, thus there may be nuanced differences in their approaches to complex periprosthetic fracture fixation. Nonetheless, we believe this increases the generalizability of our study to other multi-surgeon centers. Our two groups also differed in size, yet were statistically similar in baseline characteristics. Subsidence measurements, though based on prior literature protocols, were also contingent on radiologic landmarks and may be subjective between raters. Another limitation of the present study is the lack of differentiation between cemented and press-fit stem sub-types. For examples, we do not analyze composite beam stems compared to loaded taper stems or compare the six major uncemented stems sub-types to each other. However, all designs have the same principal of fixation in common: once the strong bond between the stem-cement or stem-bone interface is disrupted, a loose stem is born and may never again regain stability [43, 44].

## Conclusion

In geriatric patients with Vancouver B type periprosthetic fractures managed with ORIF, patients with an originally press fit stem may have lower mortality, lower estimated blood loss, and similar subsidence and hospital length of stays when compared to those with a cemented stem.

## Data Availability

The datasets used and/or analysed during the current study are available from the corresponding author on reasonable request; however, the data is not approved for public sharing by the IRB at this time. If requested, an IRB amendment may be made to this affect.
